# Clitoral Therapy Device for Alleviating Sexual Dysfunction After Female Genital Mutilation: Randomized Controlled Trial

**DOI:** 10.2196/43403

**Published:** 2023-04-21

**Authors:** Hend Reda Sakr, Yahia Ali Ahmed, Reham Mohamed Kamel, Reem Hamdy Abdelhady, Reham Alaa Elkalla, Mina Atef Georgui, Wael Osama Abd El-khalek, Mariam Hossam El Ebrashy

**Affiliations:** 1 Department of Physical Therapy for Women's Health Faculty of Physical Therapy Badr University in Cairo Cairo Egypt; 2 Department of Obstetrics and Gynaecology Faculty of Medicine Suez University Suez Egypt; 3 Department of Psychiatry Faculty of Medicine Cairo University Cairo Egypt; 4 Department of Surgery and Women's Health Faculty of Physical Therapy Sinai University Kantara Egypt; 5 Department of Physical Therapy for Surgery Faculty of Physical Therapy Badr University in Cairo Cairo Egypt; 6 Department of Physical Therapy for Internal Medicine and Geriatrics Faculty of Physical Therapy Badr University in Cairo Cairo Egypt; 7 Department of Basic Sciences Faculty of Physical Therapy Badr University in Cairo Cairo Egypt

**Keywords:** female genital mutilation, FGM, clitoral therapy device, CTD, Eros device, sex therapy, Female Sexual Function Index, FSFI, Middle East, psychological, sexual, women, sexual dysfunction

## Abstract

**Background:**

Female genital mutilation is considered a crime but is still practiced today in Africa and the Middle East, despite all the laws that make this procedure illegal due to the long-term physical and psychological harm it causes to women. Millions of girls and women living today have undergone genital mutilation, which involves removing the external female genitalia either partially or totally, based on the belief that it restricts feminine sexuality, thereby “saving” a girl for marriage. For girls and women, the surgery offers no health advantages. Girls’ right to control critical decisions regarding their sexual and reproductive health is violated because genital mutilation is frequently done against their will and frequently without their consent, leading to lifelong psychic trauma in addition to sexual dysfunction and lack of satisfaction due to distortion of the genitalia that threatens marital stability.

**Objective:**

To determine the effect of a clitoral therapy device on improving sexual domains in women suffering from sexual dysfunction after female genital mutilation.

**Methods:**

This study examined 80 married women aged from 20 to 45 years who were referred from the gynecology outpatient clinic of the Faculty of Medicine, Suez University, for sexual dysfunction resulting from female genital mutilation. The women were divided into 2 equal groups: the study group received a clitoral therapy device and traditional psychosexual education and were closely followed for 3 months, while the control group received only traditional psychosexual education for 3 months. The Arabic version of the Female Sexual Function Index (FSFI) questionnaire was used to assess sexual outcomes pre- and posttreatment in the 2 groups.

**Results:**

Our findings revealed a significant increase in the 6 domains of the FSFI pretreatment in both groups compared to posttreatment (*P*>.001), except the orgasm domain in the control group, which showed only a nonsignificant increase (*P*=.16).

**Conclusions:**

Clitoral therapy devices may be an effective, safe, noninvasive rehabilitation method for sexual dysfunction following female genital mutilation.

**Trial Registration:**

ClinicalTrials.gov NCT05039775; https://clinicaltrials.gov/ct2/show/NCT05039775

## Introduction

The Eros Clitoral Therapy Device (CTD; UroMetrics, Inc) represents a nonpharmacological technique to promote clitoris engorgement causing sensory nerve ending stimulation. This could be advantageous for a wide population of women suffering from dysfunction in their sexual relations, such as the ability to reach orgasm to nearly full satisfaction. It is a compact, handheld medical gadget with a vacuum-type vibrator manufactured of soft plastic with an appropriate cap size to cover the clitoris; it resembles a computer mouse [1]. It enhances blood flow to the clitoris by gently sucking the clitoris and the surrounding region. Blood is drawn into the clitoris by this suction, resulting in clitoral and later vaginal vascular engorgement, which increases vaginal lubrication and, as a result, stimulates sensory nerve endings. This enhances the female reaction by boosting blood flow to the clitoris and external genitalia, which facilitates reaching orgasm as vaginal lubrication is improved after using the device [1,2].

The anatomical basis for this orgasm-promoting platform is ultimately provided by vascular congestion of the genitalia, which leads to the physiological expression of the orgasmic experience. Before and during the application of the CTD, physiological examinations of clitoral and vaginal blood flow reveal a significant increase in blood velocity in these areas [2,3].

This clitoral engorgement contributes to female sexual arousal and satisfaction. It causes sensory and vasomotor nerve endings to fire, which helps with genital feeling and triggers somatic and autonomic reactions that promote arousal (ie, enlargement of the genitalia and lubrication) and thus orgasm, in addition to contributing to an early female sexual response, thereby boosting libido in women who have low desire due to decreased vaginal lubrication. This can be achieved during the use of Eros-CTD, as the woman can regulate the level and time of vacuum, which can be kept either constant or rapidly modulated according to her choice [3,4].

The Eros-CTD has been certified by the US Food and Drug Administration (FDA) for promoting female sexual function by improving orgasm quality (ie, the regularity of orgasm by direct clitoral stimulation) [3,5,6].

Without direct clitoral stimulation in intercourse, around one-third of all women reach orgasm, as most women report that they use clitoral and vaginal stimulation with their partners to experience orgasm during vaginal penetration; when underlying parts of the clitoris are stimulated, women are better able to raise their sexual excitement [7,8].

Regarding female sexual arousal disorder, small nonblinded investigations have demonstrated that using the Eros-CTD device increases blood flow to the pelvis, vagina, and clitoral region, which may dramatically enhance arousal, orgasm, and general satisfaction; for patients who prefer to avoid using pharmaceuticals or hormonal therapy, this procedure offers a successful, safe option [9].

In individuals with female sexual dysfunction who are free of cancer, the CTD has demonstrated great promise; among 32 participants who were included in a previous study, 20 had female sexual dysfunction and 12 did not. The patients with female sexual dysfunction reported more genital sensation, vaginal lubrication, orgasm ability, and sexual satisfaction after using the CTD for 3 months. All investigated domains showed gains in people without female sexual dysfunction, as well [10].

Individuals who have female sexual dysfunction and may benefit from Eros-CTD include victims of female genital mutilation (FGM). Most Middle Eastern women and women living in various regions of Africa refer to FGM or cutting as “sunna” or “pharaonic circumcision.” This surgical procedure has a profound impact on the lives of women and girls, as it hinders their psychological and physical health through anatomical alteration and chronic urogenital infection, resulting in loss of libido, arousability, and orgasm; therefore, it is currently considered a serious topic and has turned into a major global political issue [11]. FGM is classified into 4 types according to the World Health Organization (WHO): removing the prepuce with or without some or all of the clitoris is type I; removing the clitoris and partially or totally cutting the labia minora is type II; partial or total cutting of the external genitalia and decreasing the diameter of the vaginal opening is type III; clitoral or labial stretching or incision, cauterization of the clitoris and surrounding tissue by burning, tightening the tissues that surround the vaginal orifice, vaginal cutting, and introducing destructive materials such as herbs inside the vagina to cause bleeding to tighten the vaginal opening are examples of FGM type IV [12].

Despite the Egyptian High Court’s 1997 ban on the surgery, Egypt has the highest percentage of women who have undergone FGM in the world [13]. Nevertheless, Egyptian women believe they are entitled to sexual pleasure to the point that their husbands are unable to satisfy their wives sexually, which is highly dangerous to men in Egyptian culture [14]. FGM victims have unique medical, gynecological, obstetric, and psychological issues that physicians and medical staff are typically unable to handle, which is aggravated by the procedure’s illegality. The most common issue is sexual-function impairment, which can be caused by psychological trauma, scar tissue development, or partial nerve injury [15].

Disrupted sexual function with all types of FGM includes decreased vaginal secretion during intercourse, discomfort, decreased sexual satisfaction and desire, orgasm latency, and anorgasmia. Scarring, pain, and unpleasant memories linked to FGM can all contribute to these issues. Sexual dysfunction in men was also documented in a previous study conducted among women in Sudan, where type III FGM is the most common, due to male bodily discomfort when engaging in sexual activity, sex causing pain in the spouse, and marital conflict [16].

Many interventions based on psychotherapy, assistive technologies such as clitoral therapy devices and mechanical vibrators, and exercises for therapeutic purposes have been successfully used in the management of female sexual dysfunction with no known negative consequences [17].

Mechanical vibrators are intended to elicit clitoral engorgement for treating orgasm and arousal issues. Primary and secondary anorgasmia have been successfully treated with mechanical vibrators, particularly when they are paired with psychological counselling; clitoral vacuum engorgement devices like Eros-CTD engorge the clitoris using a gentle vacuum and work even when there are damaged blood vessels [18].

Patients using the InterStim Therapy Device, which causes stimulation of sacral nerves S2 to S4 to manage urinary incontinence, have reported enhancement of sexual arousal and orgasm after its application; this inspired its use for sexual arousal and orgasm disorder in women [19].

Other management protocols have been used to address sexual dysfunction in women, such as cognitive behavioral therapy and “simmering,” which involves reading sexual or educational literature, watching romantic and exotic media, keeping a journal about fantasies and sex, and focusing attention on sex. Another protocol is sensate focus: this additional therapy entails the couple committing to weekly sessions of love play, which involves guidelines for graduating from nongenital touch and excitement to genital play and intercourse. Pelvic floor rehabilitation is used to treat pain during sexual intercourse, as it may improve genital blood flow [20].

Also, topical medicines for managing dyspareunia have been applied to the vulvar or vaginal area, including topical lidocaine, which can be administered on a regular basis or used as postcoital analgesic. In addition, the off-label intravaginal use of topical diazepam has been reported to decrease pain during sexual intercourse [21].

Psychosexual support has been used to treat female sexual dysfunction symptoms by lowering anxiety levels and improving sexual skills through a variety of techniques, such as good communication, listening skills, emotion and perception expression, and conflict resolution [22]. Psychosexual support and sex education have already been identified as being successful for managing sexual dysfunction in both men and women. Short-term psychotherapy principles are followed in treatment, with specialists and patients working on specific concerns in an individual, couple, or group setting. Reinforcement, interpretation, challenge, cognitive reframing, and home practice are among the basic psychotherapy strategies traditionally used in sex therapy protocols [23]. Therefore, this study was designed to determine the effectiveness of Eros-CTD for treatment of female sexual dysfunction symptoms that result from FGM as a complement to traditional psychosexual education.

## Methods

### Subjects

This study included 80 married women aged from 20 to 45 years who were suffering from sexual dysfunction in more than one sexual domain (arousal disorder, orgasm disorder, or both). All participants were diagnosed with sexual dysfunction resulting from a history of type 1 FGM (clitoridectomy) [24]. The participants had sexual desire, were comfortable with the ideas of self-stimulation and psychosexual support, and were medically stable. All included participants were referred from the gynecology clinic of the Faculty of Medicine, Suez University, to the outpatient clinic of the Faculty of Physical Therapy, Badr University, located in Cairo, to receive clitoral therapy intervention and psychosexual education sessions between September 2021 and December 2021. Each participant underwent a detailed medical history assessment and an examination of the pelvis. Participants were excluded if they had metastases, bladder or bowel disorder, or major complications of any disease; a history of female sexual disease or sexual assault; or were using antidepressant medications.

### Design

**Figure 1:**
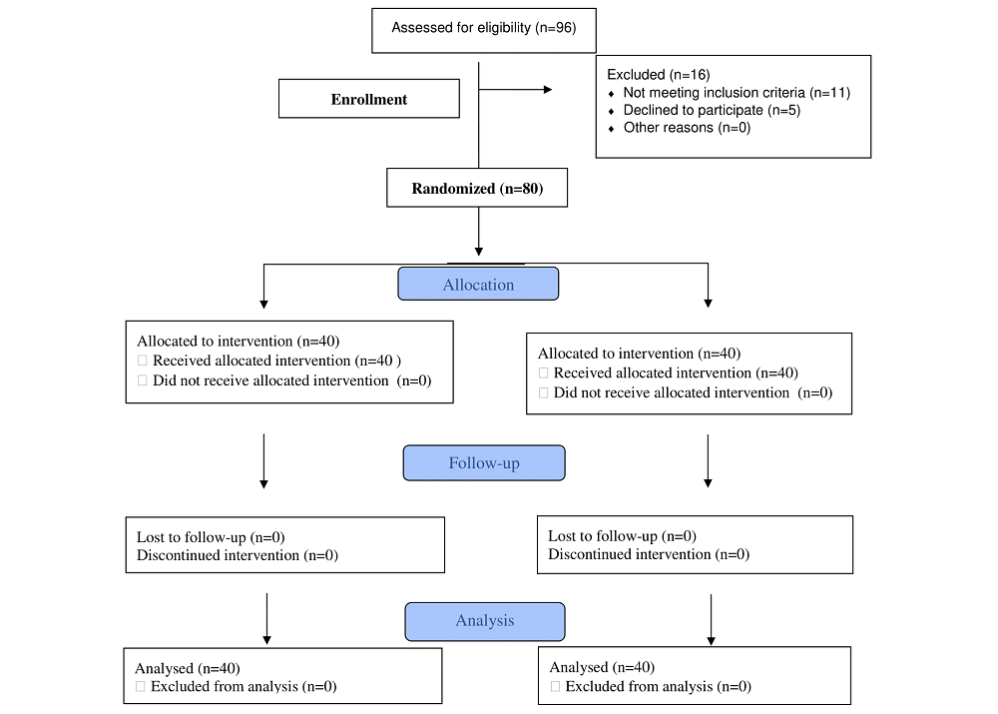
Flowchart of study design.

This was a double-blinded randomized controlled trial that used a validated, reliable questionnaire. Subjects were randomly divided into 2 equal groups in a prospective outcome registry. The design of this study is shown as a flow chart in Figure 1. A randomized computer-generated table of letters was constructed prior to the commencement of data collection by a researcher who was not involved in recruiting or managing patients. The study groups were then assigned at random using individual, sequentially lettered index cards. The index cards were folded and stuffed into opaque envelopes that were then sealed. Unaware of the baseline assessment results, a different researcher then opened these envelopes and began therapy based on the group’s task. Each participant was given either the letter A or B in the sealed envelope.

### Ethical Considerations

The study was approved by the Ethical Review Committee of the Faculty of Physical Therapy, Cairo University (P.T. REC/012/003189). This study was conducted according to the ethical guidelines of the 1964 Declaration of Helsinki and 1975 Declaration of Tokyo. It was carried out in a transparent manner, presented according to the CONSORT (Consolidated Standards of Reporting Trials) criteria, and registered at ClinicalTrials.gov (NCT05039775). After inclusion, all patients provided informed consent in the Arabic language before participation.

The privacy and confidentiality of the participants were achieved by keeping their signed informed consent forms in a locked file inside a locked locker. Participants’ personal data that were recorded on computer were kept in a secured file with a strong password that was not shared. The subjects received compensation in the form of the Eros-CTD itself and transportation to the outpatient clinic of the Faculty of Physical Therapy on buses provided by Badr University.

### Treatment

The Female Sexual Function Index (FSFI) questionnaire was used to assess all participants before and after therapy. The FSFI was created to assess sexual function in women who have engaged in sexual activity in the preceding 4 weeks. Validation research for the FSFI revealed that it had a sensitivity and specificity cutoff score of 26.55 to identify women with dysfunction in sexual activity. It has been shown to be effective in both healthy and chronically ill women [25].

The FSFI has been translated into over 20 languages, becoming the gold standard in assessing female sexual dysfunction (FSD) and an essential instrument in FSD clinical studies. The Arabic version of the FSFI is a locally approved, validated, and reliable tool for assessing FSD in the Egyptian community. Desire, arousal, lubrication, orgasm, pleasure, and pain are the 6 dimensions of FSD quantified by this 19-item, multidimensional, self-reported scale [26,27].

All participants filled out the Arabic FSFI in an examination room before starting treatment. A physiotherapist checked the questionnaires to make sure that all questions were filled in (to avoid overlooking questions). The participants were then asked to fill out the questionnaire again after 3 consecutive months of regular treatment [28].

Both groups in this study received traditional psychosexual education at the outpatient clinic of the Faculty of Physical Therapy, Badr University, under supervision of a psychiatry consultant. Psychoeducation included educating the patients and their partners on the stages of sexual arousal before orgasm and giving them tips and techniques to be applied at home based on the work of Masters and Johnson; this protocol, which depends on masturbation, is still the most common way of treating sexual problems and the most effective treatment to date for lifelong lack of orgasm in women. Patients were encouraged to gradually follow certain steps: first, to stroke the full body outside the genital areas; second, to learn how to change between active and passive positions and massage the body and genital areas using hand stimulation; and third, the woman inserted the penis into the vagina and the couple experimented with different sex positions. The participants applied these steps and returned weekly with their feedback to the therapist [10,22,28].

In addition, participants in the study group used the Eros-CTD and were closely followed for 3 months. A female physiotherapist gave direction on the use of Eros-CTD therapy after enrollment. The mechanism of regulating and tuning the vacuum to the participants’ personal comfort level was explained to them before they were invited to try using the device for 5 to 10 minutes in the examination partition. The female physiotherapist returned to the partition after this quick practice session to answer any queries and undertake a quick external genital assessment. Participants were instructed to apply the equipment alone or with a partner in the privacy of their own house. Participants modified the vacuum intensity after applying the equipment to the clitoris for a duration based on their comfort and arousal throughout the first 3 home sessions. They repeated the vacuum application 4 times weekly for 3 consecutive months for a total of 5 to 15 minutes of continuous application or 30 minutes of intermittent application. Each participant was asked to record any changes in sexual experience, such as labial engorgement, orgasm, and lubrication, during the first 3 sessions. Participants were contacted by phone to discuss their progress and any positive or negative changes [2].

### Statistical Analysis

The sample size was calculated based on pilot study conducted with 16 subjects. We estimated that a minimum proper sample size of 40 subjects in each group was necessary to reject the null hypothesis with 80% power at the α=.05 level with an effect size of 0.68 using a 2-tailed Student *t* test for independent samples. Calculation of sample size was performed with G Power (version 3.0.11; Vanderbilt University). A comparison of subject characteristics between the groups was performed using an unpaired 2-tailed *t* test. All domains of the Arabic FSFI, including desire, arousal, lubrication, orgasm, satisfaction, and pain, were compared between groups with the Mann-Whitney *U* test; within-group pre- and posttreatment comparisons were made with the Wilcoxon signed rank test. The significance level was set at *P*<.05 for all statistical tests. SPSS (version 26; IBM Corp) was used for statistical analysis in this study.

## Results

### Demographics

The Levene test for equality of variance was performed and showed that data were normally distributed. Table 1 shows participant characteristics for both groups. There were nonsignificant differences between the groups in mean age, weight, height, and BMI (*P*>.05).

**Table 1:** Participant characteristics.

	Study group, mean (SD)	Control group, mean (SD)	Mean difference	*t* (*df*)	*P* value
Age (years)	33.07 (7.21)	32.92 (7.27)	0.40	0.24 (78)	.81
Weight (kg)	61.34 (5.40)	59.72 (4.36)	1.62	1.47 (78)	.14
Height (cm)	162.93 (5.35)	163.70 (4.54)	–0.63	–0.56 (78)	.57
BMI (kg/m^2^)	22.78 (1.17)	22.44 (1.04)	0.33	1.36 (78)	.18

### Effect of Treatment on All Domains of the Arabic FSFI

#### Within-Group Comparisons

There was a significant increase in all 6 domains of the Arabic FSFI from pre- to posttreatment in both groups (*P*>.001), except in the orgasm domain in the control group, which showed a nonsignificant increase compared with pretreatment (*P*=.16), as presented in Table 2.

#### Between-Group Comparisons

There were nonsignificant pretreatment differences between groups (*P*>.05). A posttreatment comparison of the study and control groups indicated a significant rise in all domains of the Arabic FSFI in the study group compared with the control group (*P*<.05), as presented in Table 2.

**Table 2:** Within-group and between-group comparison of the Arabic version of the Female Sexual Function Index domains.

		Study group	Control group	*U*	*P* value
**Desire domain**					
	Pretreatment score, median (IQR)	1.2 (1.2-2.4)	1.8 (1.2-2.4)	–0.445	.66
	Posttreatment score, median (IQR)	4.8 (3.6-4.8)	1.2 (1.2-2.4)	–7.645	<.001
	*Z*	–5.430	–2.236		
	*P* value	<.001	.03		
**Arousal domain**					
	Pretreatment score, median (IQR)	0 (0-1.2)	1.2 (0-1.2)	–1.279	.20
	Posttreatment score, median (IQR)	3.6 (2.4-3.6)	0 (0-1.2)	–7.281	<.001
	*Z*	–5.455	–2.449		
	*P* value	<.001	.01		
**Lubrication domain**					
	Pretreatment score, median (IQR)	1.2 (0-1.2)	1.2 (0-1.2)	–0.224	.82
	Posttreatment score, median (IQR)	4.8 (3.6-4.8)	0 (0-1.2)	–7.902	<.001
	*Z*	–5.670	–2.236		
	*P* value	<.001	.03		
**Orgasm domain**					
	Pretreatment score, median (IQR)	0 (0-0)	0 (0-0)	0.0	>.99
	Posttreatment score, median (IQR)	1.2 (1.2-3.6)	0 (0-0)	–7.806	<.001
	*Z*	–5.431	–1.414		
	*P* value	<.001	.16		
**Satisfaction domain**					
	Pretreatment score, median (IQR)	0 (0-1.2)	0.6 (0-1.2)	–0.668	.51
	Posttreatment score, median (IQR)	3.6 (2.4-3.6)	0 (0-1.2)	–7.458	<.001
	*Z*	–5.601	–2.00		
	*P* value	<.001	.046		
**Pain domain**					
	Pretreatment score, median (IQR)	0 (0-1.2)	1.2 (0-1.2)	–0.315	.75
	Posttreatment score, median (IQR)	3.6 (2.4-3.6)	0 (0-1.2)	–7.749	<.001
	*Z*	–5.586	–2.236		
	*P* value	<.001	.03		

## Discussion

### Principal Findings

The purpose of this study was to determine the effectiveness of the Eros-CTD as a supplement to traditional psychosexual education on female sexual dysfunction resulting from FGM among married women in Egypt. Our findings illustrated that when compared to traditional sex therapy alone, this electrical modality may have a substantial effect on all sexual-function elements. There was a significant rise in the 6 domains of the Arabic FSFI from pre- to posttreatment (*P*>.001), except the orgasm domain in the control group, which showed a nonsignificant increase compared to pretreatment (*P*=.16).

### Comparison to Prior Work

The study population was selected with reference to a prior study, conducted in 2003, of 7 patients with sexual arousal disorder who had normal hormone levels at the time of the study. The 7 respondents were able to use the equipment with ease and noted minor to modest pleasure and being able to reach orgasm at home with no negative side effects. That study revealed that using the Eros-CTD induced a significant increase in the diameter of the clitoral and corpus spongiosum and increases in the peak systolic and end-diastolic velocity values in the clitoral and corpus spongiosum; all factors improved in the orgasm domain, in agreement with the results of our study [29].

In another previous study, conducted by Schroder et al [2], patients with sexual dysfunction induced by radiation therapy used the Eros-CTD 4 times weekly for 3 consecutive months for the purpose of self-stimulation and passion foreplay for 15 to 30 minutes intermittently. Improved vaginal mucosal coloration, hydration, and elasticity were found during gynecologic exams after 3 months of treatment, showing enhancement in all aspects assessed by the sexual function assessment instruments; this also supports the results of our study [30,31].

To ensure the safety of the therapy, we chose the Eros-CTD manufactured by Uro-Metric, because it is approved by the FDA and is intended to promote excitement by gently suctioning blood flow to the clitoris. Two earlier short-term trials found that CTDs help women with sexual arousal disorder and decreased lubrication, and hence may reduce dyspareunia linked with diminished desire, corroborating the findings of this study [6,32-34].

Another study, conducted among 57 women with spinal cord injuries resulting in altered sexual response and decreased sexual arousal, examined the effect of vibratory stimulation on arousal as measured by the pulse amplitude of the vagina. Forty-six women with spinal cord injury and 11 nondisabled women in a control group were included. In both groups, stimulation of the clitoris by vibration resulted in higher pulse amplitude of the vagina as compared to manual clitoral stimulation, which corresponds to the findings of this study [35].

Among studies of different types of disorders, a previous study conducted among women with diabetes and arousal or orgasm disorders concluded that they could benefit from the FDA-approved Eros-CTD, as it increased genital blood circulation and improved the sensitivity of the genitalia; these results also confirm our study’s findings [36].

Women with hypoactive sexual desire disorder can use nonpharmacological modalities like sex psychotherapy, vaginal dilators, and, if they have arousal or orgasm disorder, Eros-CTD equipment, according to the recommendations of the Association of Reproductive Health Professionals, which supports the idea of using these modalities with FGM patients [37].

Previous studies have shown that sex therapy, in comparison with other forms of psychosexual support therapy, appears to be more effective and faster for treating various problems related to sexual function and life and had a positive impact. However, some of the past literature does not show that sex therapy is effective for all sexual disorders observed in therapeutic settings; this corresponds to the outcomes of our study to some extent, as the control group who only received sex therapy showed improvement in all sexual domains except orgasm [22].

A previous study conducted among women with multiple sclerosis who had sexual dysfunction due to neural defects, decreased self-confidence, and depression revealed that these patients were sensitive to clitoral vibration, suggesting clitoral vibrators can be used for diagnosing sexual dysfunction in women with multiple sclerosis; this may be due to the vibratory sensation being mediated by large diameter nerve fibers that connect from the periphery to the center through the dorsal columns, which serve as the natural mediators of sexually induced sensations. This supports and explains the results of this study and the effectiveness of the Eros-CTD in improving sexual domains in women with sexual dysfunction due to FGM [38].

Another study conducted among 19 women who used clitoral vibration for the first time in their lives (once weekly for 1 month) found changes in the pattern of orgasm, supporting the idea that the Eros device can improve orgasm, which agrees with the results of this study [39].

### Strengths

This study had many strengths. First was the availability of a valid and reliable Arabic questionnaire to assess the outcomes of the study. Second was the availability of a validated and reliable portable Eros device that is FDA approved. Third, the data were easily analyzed, as they were precise. Fourth, the selection process was well designed, and we selected subjects from different reproductive ages so that we could obtain a representative sample of the population, which made our findings generalizable.

### Limitations

The first limitation of this study was the restricted sample size and sample availability; although FGM is a very common procedure among Arabic women, these women often do not have enough courage to face the resulting sexual problems and discuss them with the appropriate specialists. This is because of old Eastern traditions and customs that place blame on married women who discuss or complain about any sexual problems in their relationship with their husband. Second, there was uncertainty among our participants on how to correctly practice psychosexual education during intimate relations.

### Conclusions

The results obtained in this study lead us to conclude that CTDs may be a safe, effective, reasonably priced modality that can be used to enhance sexual domains in women who suffer from sexual dysfunction in one or more sexual domains as a result of FGM.

## Appendix

Multimedia Appendix 1 CONSORT checklist.

## Abbreviations

CTD: clitoral therapy device
FDA: Food and Drug Administration
FGM: female genital mutilation
FSFI: Female Sexual Function Index
WHO: World Health Organization

## Data Availability

The data sets generated during and/or analyzed during the current study are not publicly available to ensure the privacy of our patients, but are available from the corresponding author on reasonable request.
