# Supplementary material for: Clitoral Therapy Device for Alleviating Sexual Dysfunction After Female Genital Mutilation: Randomized Controlled Trial
Source: JMIR Rehabil Assist Technol. 2023 Apr 21;10:e43403. doi: 10.2196/43403 (PMC10163396; doi:10.2196/43403)
Supplement: Multimedia Appendix 1 [file rehab_v10i1e43403_app1.pdf]

Reporting checklist for randomised trial.

Based on the CONSORT guidelines.

	Reporting Item	Page Number
**Title and Abstract**		
Title	Clitoral Therapy Device in Alleviating Sexual Dysfunction After Female Genital Mutilation: a randomized controlled trial.	1
Abstract	**Background:** Female genital mutilation is considered a crime that is still practiced today in Africa and the Middle East, despite all the laws that make this procedure illegal due to the long-term physical and psychological harm it causes to women. Millions of girls and women alive today have encountered genital mutilation by removing external female genitalia either partially or totally, believing that it restricts feminine sexuality; ‘saving’ a girl for marriage. For girls and women, the surgery offers no health advantages. Girls' right to control critical decisions regarding their sexual and reproductive health is violated because it is frequently done against their will and frequently without their consent; leading to psychic trauma that lasts for her whole life in addition to sexual dysfunction and lack of satisfaction due to genitalia distortion that threaten the stability of her marriage. **Objective:** to find out the influence of clitoral therapy device on improving sexual domains in women suffering from sexual dysfunction after female genital mutilation. **Methods:** Eighty married females, with age range from 20 to 45 years old referred from the Gynecology outpatient clinic of Faculty of Medicine, Suez University with sexual dysfunction resulting from female genital mutilation and were divided into two equal groups. Study group received Clitoral Therapy Device and traditional psychosexual education and were closely followed for three months, while control group received	1

traditional psychosexual education only for three months. Arabic Female Sexual Function Index Questionnaire (FSFI) assessed sexual outcomes pre- and post- treatment in the two groups**. Results:** Findings revealed a significant increase in the six domains of Arabic Female Sexual Function Index post-treatment in both groups compared with that pre-treatment (p<.001) except orgasm domain in control group; there was non-significant increase with that pre-treatment (p>.05). **Conclusions:** Clitoral therapy device may be effective, safe, non-invasive rehabilitation method of sexual dysfunction that followed female genital mutilation.
Keywords: Female genital mutilation (FGM); clitoral therapy device (CTD); EROS device; Sex therapy; female sexual function index (FSFI).

Introduction

Background and objectives	Most Middle East women and females living in various regions of Africa refer to their female genital mutilation or cutting as Sunna or Pharaonic circumcision which has a profound impact on women and girls' lives, as it hinders their psychological and physical health through anatomical alteration and chronic urogenital infection, resulting in loss of libido, arousability, and orgasm so it is considered a serious topic right now that has turned into a major global political issue……Clitoral engorgement contributes to female sexual arousal and satisfaction as it causes sensory and vasomotor nerve endings to fire, which help with genital feeling, orgasm by triggering somatic and autonomic reactions that promote arousal (enlargement of genitalia and lubrication) and thus orgasm, in addition to its involvement in early female sexual response, boosting libido in women who have low desire due to decreased vaginal lubrication. The EROS-CTD is a non-pharmacological technique that promotes clitoris engorgement causing sensory nerve ending stimulation. It can be advantageous to a wide population of females suffering from dysfunction in her sexual relation reaching orgasm to nearly full satisfaction. The EROS-CTD equipment is applied on clitoris and pumping action is operated forming	1, 2, 3

	mild vacuum on clitoris causing clitoral engorgement. The current trial was therefore set to find out the effect of CTD associated with psychosexual education on the sexual domains in females with history of genital mutilation.	
Background and objectives	In the current study we tested the hypothesis that using clitoral therapy device in addition to psychosexual support after female genital mutilation would improve sexual domains including desire, orgasm, arousal, lubrication, satisfaction and pain.	1
**Methods**		
Trial design	This was parallel group, randomized controlled trial with double blinded, balanced randomization 1:1, conducted in Egypt.	3
Trial design	N/A	
Participants	Eligible participants were all married women with the age ranging from 20 to 45 years and were suffering sexual dysfunction in more than one sexual domain (arousal and/or orgasm disorder). All participants were diagnosed with sexual dysfunction resulting from history of female genital mutilation surgery type 1, they can have sexual desire, comfortable with the idea of self-stimulation, psychosexual support and medically stable. Excluded participants were patients with metastases, bladder or bowel disorder, major complications of any disease, or history female sexual disease, sexual assault or under anti-depressant medication.	3
Participants	All participants included were referred from the Gynecology Clinic of Faculty of Medicine, Suez University to the outpatient clinic of Faculty of Physical therapy, Badr University in Cairo to receive clitoral therapy intervention and psychosexual education sessions, between September 2021 to December 2021.	3
Interventions	Both groups in this study received traditional psychosexual education at outpatient clinics of Faculty of Physical Therapy, Badr university under supervision of a psychiatry consultant,	4, 5

	psychoeducation included educating the patients and their partners the stages of sexual arousal till orgasm, and giving them tips and teaching techniques to be applied at home based on Masters and Johnson technique. Patients were encouraged to follow certain steps gradually first to stroke the full body without genital areas, then to learn changing positions between active and passive and massaging the body and genital areas, using hand stimulation, then the woman entering the penis to the vagina and experimenting different sex positions. Applying in steps and returning with their feedback to the therapists weekly. In addition, participants in the study group used clitoral therapy device and were closely followed for three months. A female physiotherapist gave the direction of use EROS-CTD therapy after enrollment. Participants were told the mechanism of regulation and tuning vacuum to their personal comfortability before being invited to try using the device for 5 to 10 minutes in the examining partition. The female physiotherapist returned to the partition after this quick practice for answering any queries and undertake fast external genital assessment. Participants were instructed to apply the equipment alone or with a partner in the privacy of their own houses. Participants modified the vacuum intensity after applying the equipment to the clitoris for duration based on their comfort and arousal throughout the first three home sessions. They repeated vacuum application four times weekly for three consecutive months, for a total of 5 to 15 minutes of continuous application or 30 minutes of intermittent application. Each participant was asked to record any changes in sexual experience, such as labial engorgement, orgasm, and lubrication during the first three sessions.	
Outcomes	Female Sexual Function Index Questionnaire FSFI was used to assess all participants before and after therapy. Desire, arousal, lubrication, orgasm, pleasure, and pain are among the six dimensions of female sexual dysfunction (FSD) quantified by this 19-item multidimensional self-reporting scale. All participants filled the Arabic Female Sexual function index FSFI in an examining room before starting	4

	treatment. The physiotherapist checked to make sure that all questionnaire points were filled in (to avoid overlooking questions). Then they asked to fill the questionnaire after three consecutive months of regular treatment.	
Outcomes	N/A	
Sample size	The sample size was calculated based on pilot study conducted on 16 subjects. We estimated the minimum proper sample size as 40 subjects in each group that can be able to reject the null hypothesis with 80% power at $\alpha = 0.05$ level and effect size = 0.68 using Student's t test for independent samples.	5
Sample size	N/A	
Randomization - Sequence generation	A randomized table of letters generated by computer, was constructed prior to the commencement of collecting data. And ever newly participant was enrolled after picking a card with a letter randomly, then enrolled into group.	
Randomization - Sequence generation	Fixed allocation randomization was applied to the participants throughout the study, distributing them among study and control groups according to the chosen letter.	
Randomization - Allocation concealment mechanism	A randomized table of letters generated by computer, was constructed prior to the commencement of collecting data through a researcher who wasn't a member in recruiting and/or managing patients. The treatment groups were assigned at random using individual and sequentially lettered index cards. The index cards were pleated and stuffed into invisible envelopes that were sealed.	3
Randomization - Implementation	A researcher who wasn't a member in recruiting and/or managing patients generated the allocation sequences. Another therapist opened envelopes and began enrollment based on the group's task.	3
Blinding	Participants were blinded to which group they were assigned	3

	into. The psychiatrist who offers the psychosexual therapy was blinded to which group participants were assigned into and the female physiotherapist was blinded to the groups of participants upon assessment of outcomes.	
Blinding	N/A	
Statistical methods	The comparison of subject characteristics between groups was performed using the unpaired t-test. All domains of Arabic Female Sexual Function Index including (Desire, Arousal, Lubrication, Orgasm, Satisfaction and Pain) were compared between groups by Mann–Whitney U test and were compared between pre-and post-treatment in each group by Wilcoxon Signed Ranks. Regarding all statistical tests, the significance level was set at *p* .05. Statistical package for social studies (SPSS) version 26 for windows (IBM SPSS, Chicago, IL, USA) was used for statistical analysis in this study.	5, 6, 7
Statistical methods	Levene's test for equality of variance was performed showing that data was normally distributed.	5
**Results**		
Participant flow diagram (strongly recommended)	A flow diagram shows eighty subjects were divided into two equal groups in a random way to engage into this prospective outcome registry as shown in figure 1.	
Participant flow	96 subjects were eligible, 16 subjects were excluded, 11 of them didn't meet criteria of selection and 5 declined to participate in the study.	
Recruitment	Recruitment started on September 2021, and they attended to the clinic at randomization (baseline) then at three months interval.	3
Recruitment	N/A	
Baseline data	Available	
Numbers analyzed	For each group, 40 participants were recruited. The study group received clitoral therapy device in addition to psychosexual support, while control group received	5

	psychosexual support only.	
Outcomes and estimation	Available in table 2	
Outcomes and estimation	N/A	
Ancillary analyses	N/A	
Harms	N/A	
**Discussion**		
Limitations	The present study was restricted by sample size and sample availability; although FGM is very common procedure among Arabic female, but these females haven't the enough courage to face the resulting sexual problems and discussing them with the appropriate specialists. This is because of the old eastern tradition and customs that blame on the married woman when she discusses or complains from any sexual problem in the relation with her husband. In addition to the uncertainty of practicing the psychosexual education correctly during the intimate relation.	9
Generalizability	The results of a study are broadly applicable to all females who are suffering from sexual dysfunction whatever the cause is, so the results of this study have good generalizability.	
Interpretation	Available at discussion section.	7, 8
Registration	The study was registered in clinical trial.gov with ID: (NCT05039775) and named Hend3.	4
**Other information**		
Interpretation	N/A	
Registration	The trial is registered at ClinicalTrials.gov, number NCT05039775.	

Protocol	N/A
Funding	N/A
